# Conserved gene clusters in entomopathogenic filamentous fungi

**DOI:** 10.1590/1678-4685-GMB-2025-0168

**Published:** 2026-04-17

**Authors:** Alexandra de Azevedo da Rocha, Charley Christian Staats

**Affiliations:** 1 Universidade Federal do Rio Grande do Sul, Programa de Pós-Graduação em Biologia Celular e Molecular, Centro de Biotecnologia, Porto Alegre, RS, Brazil.

**Keywords:** Entomopathogenic fungi, secondary metabolites, biosynthetic gene clusters

## Abstract

Entomopathogenic filamentous fungi from the Order *Hypocreales* are studied for biological pest control due to their ability to infect arthropods. Their biotechnological potential lies in the production of secondary metabolites (SMs), encoded by biosynthetic gene clusters (BGCs), which are crucial for the fungal life cycle and infection. Due to the different roles played by SMs in fungi, the in-depth study of these molecules has increased over the last years. Considering the proven biotechnological importance of species of the genus *Beauveria* have already shown, we performed an *in-silico* analysis of BGCs of the species from the order *Hypocreales* to uncover potential conserved pathways and find new candidates for biological pest control. A total of 295 genome sequences were analyzed using antiSMASH, allowing the identification of 12,968 BGCs. Conservation analysis was performed using BiG-SCAPE, which could group these BGCs into 2,127 biosynthetic gene families. Despite the presence of conserved gene clusters, especially within the same genus, when the comparison of BGCs was performed within the order, a large part of them is orphaned, highlighting the great diversity of BGCs and consequently the chemodiversity of fungal species. Our approach helps to uncover new molecules that may provide new biotechnological tools.

## Introduction

The order *Hypocreales* (phylum Ascomycota) encompasses a broad spectrum of fungal lineages characterized by remarkable ecological plasticity and significant anthropogenic impact. These fungi undergo extensive genomic reconfiguration as an adaptive response to niche availability. Notably, approximately 40% of the genes previously categorized as unique within this order possess homologs in other fungal taxa, suggesting that the perceived novelty of “lineage-specific” genes may be overestimated (Wu and Cox, 2021). Furthermore, the adaptation to novel environments and the transition between complex lifestyles appear to be facilitated by a relatively parsimonious set of essential genes. Consequently, it is hypothesized that the entomopathogenic members of *Hypocreales* followed an evolutionary trajectory originating from endophytic or phytopathogenic ancestors (Wang and Wang, 2017; St Leger, 2024).

Entomopathogenic fungi (EPF) are cosmopolitan organisms known for their association with plants and for infecting arthropod hosts. These fungi hold immense biotechnological potential, as they are globally employed in biological pest control (Hong et al., 2024; Alviti Kankanamalage et al., 2025; de Sousa et al., 2025). To successfully infect arthropods hosts, EPFs produce an array of virulence determinants. These molecules primarily function to disrupt the host cuticle and inactivate host defenses (Qin et al., 2023; de Miranda et al., 2024; Ying, 2024). Additionally, these fungi produce a vast repertoire of secondary metabolites (SMs), which are small-molecule compounds that play crucial roles in their ecological interactions and during fungal infection, even though these metabolites are not generally essential for fungal survival (Zhgun, 2023; Shahbaz et al., 2024; Jinna et al., 2025). 

The discovery of secondary metabolites (SMs) and their pivotal role in the development of life-saving antibiotics, such as penicillin, has catalyzed a steady increase in mycological research (Walton, 2000). For decades, a fundamental correlation has been observed between the synthesis of natural products and fungal morphological development, carrying profound implications for genetic regulation and evolutionary biology (Keller et al., 2005). The biosynthesis of SMs is regulated by intricate regulatory networks and is widely recognized to be orchestrated by a diverse set of proteins encoded by genes clustered into biosynthetic gene clusters (BGCs). These gene clusters are organized as contiguous regions within the genome and generally harbor all elements for the synthesis of specific SMs (Zhgun, 2023). BGCs typically contain backbone genes whose encoded protein domains serve as informative markers for inferring the class of secondary metabolites produced, such as nonribosomal peptide synthetases (NRPS), polyketide synthases (PKS), ribosomally synthesized and post-translationally modified peptides (RiPPs), terpenes, hybrid molecules, and others (Xu et al., 2023).

Considering the ongoing expansion of fungal genome sequences available, it is feasible to assume that an increasing number of BGCs, paving the way for the discovery of promising new bioactive secondary metabolites with valuable applications (Gu et al., 2020; Riedling et al., 2024). The Ascomycota phylum harbors a diverse array of filamentous fungi with significant ecological and economic importance. Given that the primary source of their remarkable biotechnological potential lies in the production of SMs (Keller, 2019; Zhgun, 2023), the conservation analysis of BGCs in entomopathogenic filamentous fungi is crucial for uncovering new bioactive compounds and identifying promising candidates for biological pest control (Pedrini, 2018). Accordingly, we focused on the order *Hypocreales*, a rich source of entomopathogenic fungal species, employing a refined *in silico* approach to search for conserved genes of interest and novel bioactive compounds derived from BGCs with potential applications in agriculture.

## Material and Methods

### Genome sequences of *Hypocreales*

A total of 295 genome sequences from *Hypocreales* were retrieved from the National Center for Biotechnology Information (NCBI). Genome database using the NCBI datasets (v14.27.0 (O’Leary et al., 2024)) tool (Table S1). All annotated genomes of the order *Hypocreales* were retrieved and stored as the annotated genome (gbff) files. Completeness of genome sequences was evaluated using BUSCO v6 (Tegenfeldt et al., 2025) with genome mode and the hypocreales_odb12 dataset.

### Identification of biosynthetic gene clusters and gene clusters families in *Hypocreales* genomes

The annotated genome gbff files were used for BGC prediction using the antiSMASH v6.1.1 webserver (Blin et al., 2021) with the following options activated (KnownClusterBlast, ClusterBlast, MIBiG cluster comparison) and the Detection strictness set to relaxed. The resulting gbk files were then used for gene clusters families (GCFs) prediction using the BiG-SCAPE tool v 1.1.2 (Navarro-Muñoz et al., 2020). Summarization of total BGCs, GCFs, as well as GCFs size are performed using custom R scripts, available at ZENODO (https://doi.org/10.5281/zenodo.15307303). Fine structure conservation of selected BGCs were further analyzed using the CAGECAT server (van den Belt et al., 2023). 

### Expression analysis of selected clusters

The Prefetch tool from the SRA Toolkit v 3.0.0 (https://github.com/ncbi/sra-tools) was used to retrieve sequencing data from the NCBI Sequence Read Archive (SRA) files available on the NCBI for detailed analysis. Runs from the NCBI Bioproject codes PRJNA574771 (Transcriptome sequencing of *Acyrthosiphon pisum* by infecting with *Beauveria bassiana*) and PRJNA1032759 (A dual RNA-seq approach to study oral infection of the red flour beetle *Tribolium castaneum* with the entomopathogenic fungus *Beauveria bassiana*) were retrieved and converted to fastq files using the fasterq-dump with the “--split-files’’ option. Fastp (Chen et al., 2018) was then used to clean and quality-filter the raw fastq data for further analysis. Processed RNA-seq reads were aligned to *B. bassiana* ARSEF 2860 genome sequence (NCBI accession code GCA_000280675.1) using the STAR tool with the “--quantMode” option to generate a matrix of read counts per gene. DESeq2 package was used to obtain the normalized expression of each gene in the distinct conditions, which was used for the construction of heatmaps with the ‘Pheatmap’ package. The R scripts are also available at Zenodo (https://doi.org/10.5281/zenodo.15307303).

## Results

### Identification of BGCs in *Hypocreales* genome sequences

**Figure 1 -:**
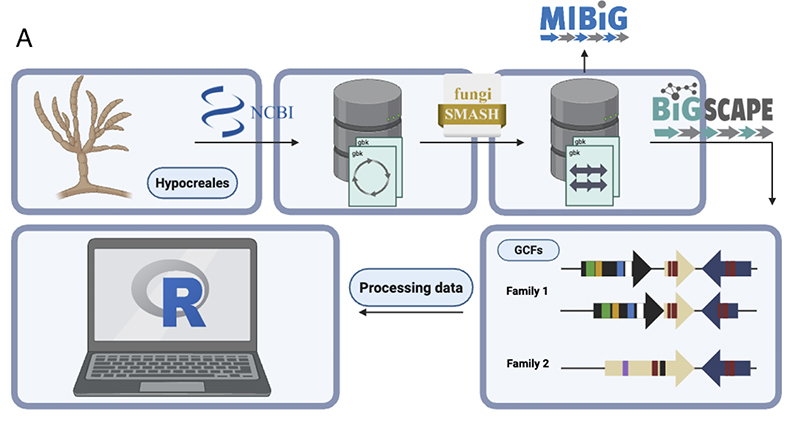
Schematic illustration of the methodology for inferring (A) BGCs and GCFs in hypocrealean genomes.

**Figure 2 -:**
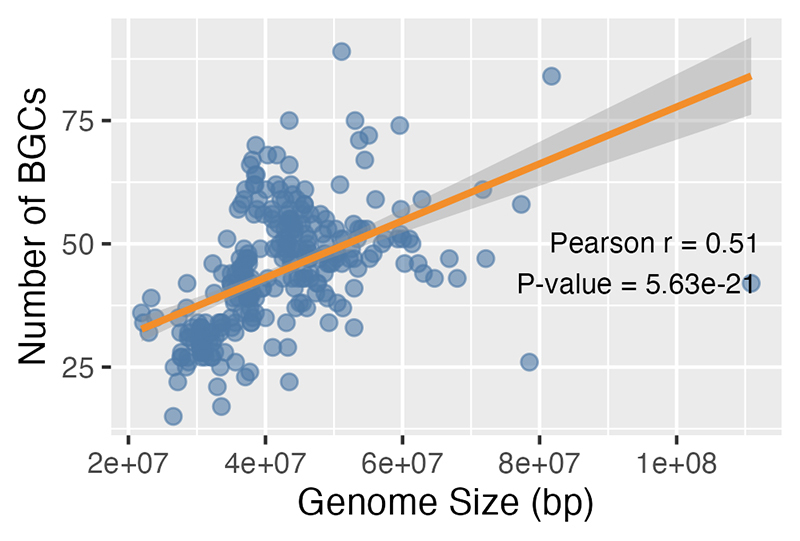
Genome size does not correlate with the number of biosynthetic gene clusters (BGCs) in Hypocrealean genomes. Each dot represents a fungal genome, plotted by its total genome size (Mb) and the number of predicted BGCs. A linear regression model was fitted to assess the relationship between genome size and BGC count. The Pearson’s correlation coefficient (r) and *P*-value were calculated using a simple linear regression analysis.

We recovered a total of 295 genomic sequences from fungal isolates of the order *Hypocreales* available in NCBI, as of February 2022. To infer the BGCs and analyze their conservation, a stringent pipeline (Figure 1) was constructed and applied for each of these genome sequences. Analysis with antiSMASH resulted in a total of 12,968 BGCs distributed along the genomic sequences. A slight positive correlation between the genome size and the number of inferred BGCs was observed (Figure 2), indicating that larger genomes tend to harbor more BGCs, though other evolutionary factors also drive BGC count in the genomes analyzed. Such gene clusters were then categorized based on the class of metabolite they are predicted to produce, following the classification proposed by BiG-SCAPE: Non-Ribosomal Peptides (NRPS), Polyketides (PKSI and PKSOther), Terpenes, Ribosomally synthesized and post-translationally modified Peptides (RiPPs), Hybrids (PKS-NRP_Hybrids), and Others. A total of 4,950 BGCs were classified as NRPS, representing the largest group. PKS accounted for 2,899 BGCs, followed by 2,314 Terpenes, 1,398 Others, 1,156 Hybrids, 211 PKSother, and 40 RiPPs, the smallest group.

**Figure 3 -:**
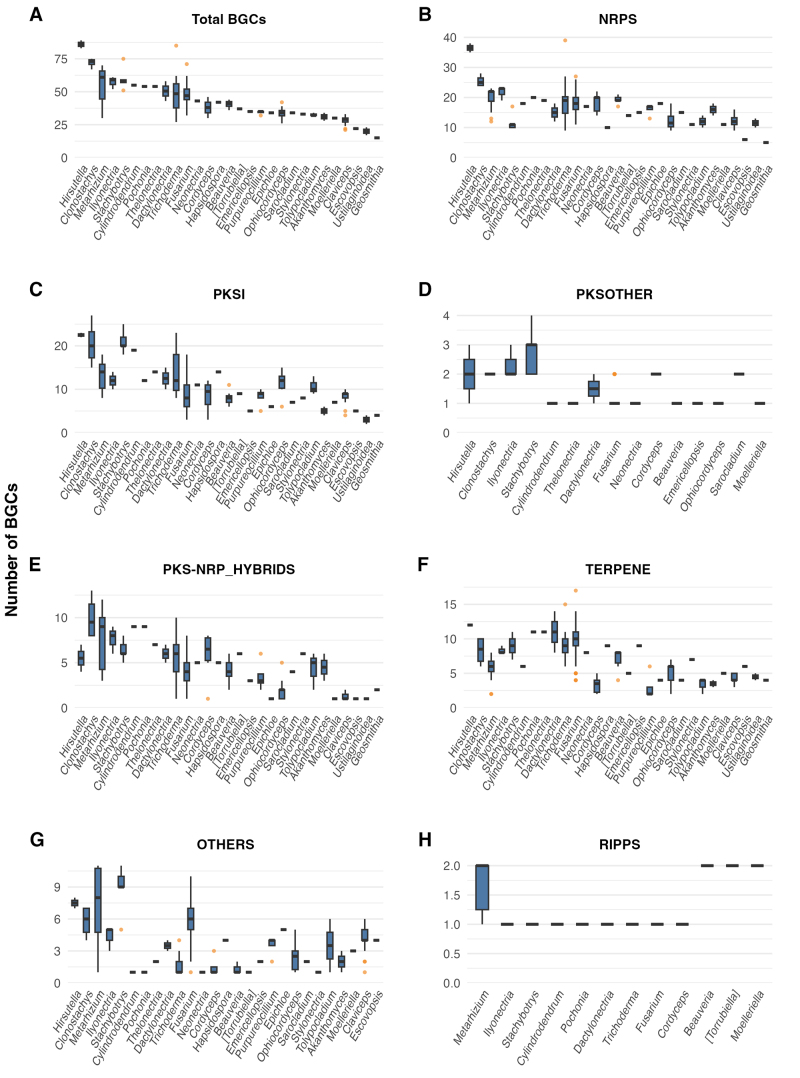
Distribution and abundance of predicted biosynthetic gene clusters (BGCs) across fungal genera in *Hypocreales*. The number and types of predicted biosynthetic gene clusters (BGCs) identified in the genomes of various fungal species, grouped by genus, within the order *Hypocreales* and related taxa. Data are presented as box plots, where the box represents the interquartile range (IQR), the horizontal line within the box indicates the median, and the whiskers extend to 1.5 times the IQR from the box edges. Panels (A) through (H) show the distribution for different BGC classes: (A) Total BGCs, (B) NRPS, (C) PKSI, (D) PKSOTHER, (E) PKS-NRP HYBRIDS, (F) TERPENE, (G) OTHERS, and (H) RIPPS.

The distribution of BGCs along the analyzed genomes was further evaluated. For the four genera containing the higher number of species with genome sequences available (*Claviceps, Fusarium*, *Trichoderma*, and *Metarhizium*), we could detect a large variation in the number of BGCs in each genome (Figure 3). This result was even observed with completeness of the genome estimated to be high as revealed by BUSCO analysis (all genomes analyzed displayed over 95% of complete BUSCOs). For instance, there is a variation from 15 BGCs in *Geosmithia morbida* to 89 BGCs in *Hirsutella minnesotensis*, and an average total BGCs per genome of 48.77. Globally, NRPS and PKSI are the most abundant BGC classes, followed by Terpene and PKS-NRP_HYBRIDS. PKSOTHER and RIPPS are the least common. 

### Conservation analysis of GCFs in *Hypocreales* genomes

**Figure 4 -:**
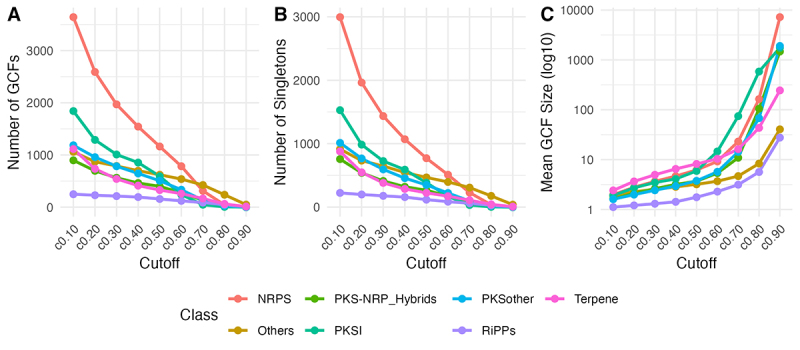
Determination of cutoff value for assignment of BGCs to GCFs. Rarefaction curves were constructed using distinct cutoff values, and the number of GCFs (A), Singletons (B), as well as the average GCF size (C) were determined. Cutoff 0.6 was selected for its optimal balance between sensitivity and specificity for proper BGC classification.

To evaluate the conservation patterns of the predicted BGCs across the genomes, the antiSMASH output files were further analyzed using BiG-SCAPE (Navarro-Muñoz et al., 2020). BiG-SCAPE employs distance metrics based on the presence and order of conserved protein domains within predicted BGC products to group similar clusters into Gene Cluster Families (GCFs). This analysis was performed independently for BGCs classified into the following categories: NRPS, Others, PKS-NRP Hybrids, PKSI, PKSother, RiPPs, and Terpene. To determine the optimal clustering stringency, the analysis was conducted across a range of cutoff values (from 0.1 to 0.9, with increments of 0.1). The selection of the best cutoff was based on analyzing its impact on the number of resulting GCFs, the number of singletons BGCs (those not assigned to any GCF), and the mean GCF size. Visual inspection of these metrics against increasing cutoff values suggested that, in general, a cutoff value of 0.6 provided a robust clustering result for most classes. Below this threshold, increasing the cutoff led to a steady decrease in the number of GCFs and singletons as more BGCs were grouped (Figures 4A and B, respectively). However, increasing the cutoff beyond 0.6 often resulted in a more drastic increase in the mean GCF size (Figure 4 C), indicating the merging of less similar BGCs. Therefore, a cutoff of 0.6 was selected for subsequent analyses. Analysis at this cutoff reveals differential conservation patterns among BGC classes, with PKSI, Terpene, and NRPS BGCs showing greater tendencies to form larger, less singleton-heavy families compared to RiPPs and “Others.” This differential clustering reflects inherent differences in the evolutionary dynamics and conservation landscapes of these distinct secondary metabolite types.

**Figure 5 -:**
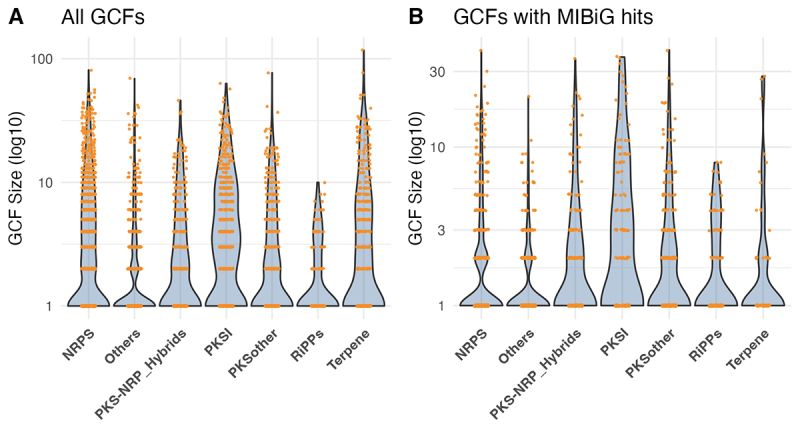
Distribution and comparison of Gene Cluster Family (GCF) sizes across biosynthetic classes at a cutoff of 0.60. (A) GCF Size Distribution of All GCFs: Size distribution for all predicted GCFs identified in the dataset. (B) GCF Size Distribution of GCFs with MiBIG Entries: Size distribution specifically for those GCFs that contain at least one BGC that is also present in the MiBIG database. Violin plots showing the distribution of GCF sizes (on a log10 scale) for each biosynthetic gene cluster (BGC) class, using the BiG-SCAPE clustering results with a cutoff value of 0.60. Orange points represent individual GCF sizes, jittered horizontally for visibility.

The size of each GCF was further evaluated. Distribution of BGC sizes by class revealed a marked heterogeneity (Figure 5A). To quantify disparities in GCF sizes across biosynthetic gene cluster (BGC) classes, we tested whether their distributions followed power-law scaling - a pattern typical of systems dominated by a few large elements among many small ones. GCF sizes were fit to discrete power-law models and compared to lognormal and exponential distributions using Vuong’s likelihood ratio test, with 1,000 bootstrap replicates used to assess parameter stability. While NRPS, PKSI, and RiPPs did not show significant differences between models (p-values 0.83, 0.48, and 0.05, respectively), all other classes (e.g., Others, PKS-NRP hybrids, PKSother, Terpene) were significantly better fit by a lognormal distribution (p < 0.02) - Table S2. These findings, particularly the lack of significant difference between power-law and lognormal fits in major classes like NRPS and PKSI, suggest that while some biosynthetic systems may exhibit heavy-tailed distributions, the overall architecture of fungal GCF sizes is more consistent with lognormal variation than with true power-law scaling. Furthermore, it is noteworthy that when accounting only for GCFs containing BGCs present in the MiBIG database, the distribution of GCF sizes decreases drastically (Figure 5B ), suggesting that the unexplored and unidentified portion of fungal chemodiversity is even greater than currently recognized.

### Identification of BGCs and GCFs in entomopathogenic fungi

**Figure 6 -:**
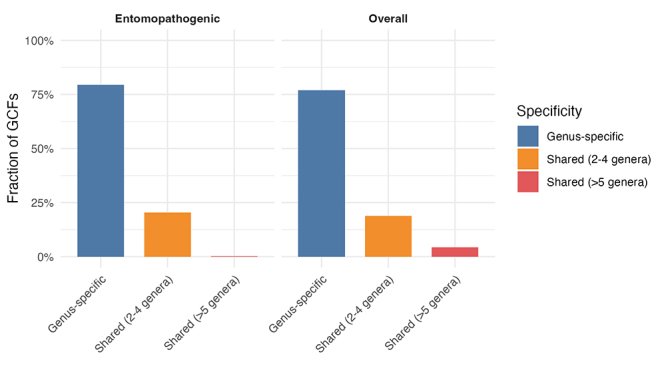
Distribution of Gene Cluster Family (GCF) taxonomic specificity in entomopathogenic and overall datasets. Bar plots show the fraction of GCFs classified according to their taxonomic specificity in entomopathogenic species (left) and across all species (right).

Entomopathogenic fungi, especially from the genera *Beauveria* and *Metarhizium*, are widely used for the biological control of agricultural pests, as well as vectors of human diseases. We hypothesized that biosynthetic gene clusters (BGCs) involved in the virulence of entomopathogenic fungi would be conserved across genera that include insect-infecting species. To test this, we first evaluated the BiG-SCAPE output to determine the proportion of gene cluster families (GCFs) that are genus-specific versus those shared among multiple genera. As expected, the majority of GCFs were genus-specific (χ² = 1888.9, df = 2, p < 2.2e-16; Figure 6). We then investigated whether this pattern also could be observed within entomopathogenic genera. For this, we filtered GCFs found in *Metarhizium*, *Beauveria*, *Cordyceps*, *Hirsutella*, *Tolypocladium*, *Purpureocillium*, and *Akanthomyces* genera, and examined their distribution. Again, the largest proportion of GCFs was genus-specific (χ² = 657.07, df = 2, p < 2.2e-16; Figure 6). 

We then analyzed the GCFs that are shared only by entomopathogenic genera. We identified a set of GCFs exclusively shared among genera traditionally associated with entomopathogenic lifestyles. In our dataset, a total of 16 GCFs were present in at least three distinct entomopathogenic genera but absent from non-entomopathogenic taxa. The most frequently co-occurring genera among these shared GCFs were *Beauveria*, *Cordyceps*, and *Akanthomyces*, suggesting conservation of specific biosynthetic pathways across these lineages. Notably, GCF_5048 was found in four genera (*Akanthomyces*, *Beauveria*, *Cordyceps*, and *Hirsutella*), indicating a potentially widespread and conserved function (Table S3). These observations are consistent with the taxonomic relationships among the genera: *Beauveria*, *Cordyceps*, and *Akanthomyces* all belong to the family *Cordycipitaceae*, while *Hirsutella* and *Purpureocillium* are members of *Ophiocordycipitaceae*, and *Metarhizium* belongs to *Clavicipitaceae*. Fine mapping and comparison of the above genera members confirmed that our approach for setting the BiG-SCAPE cutoff value to 0.6 is not too relaxed, as all genes of a BGC clearly show orthologs in the BGCs of a family Synteny analyses of the BGCs found within these families are shown in Supplementary Figures S1 -S16. These results support the hypothesis that certain BGCs are conserved among entomopathogenic fungi, potentially reflecting shared virulence strategies or ecological adaptations.

### Expression analysis of entomopathogenic fungi BGCs

**Figure 7 -:**
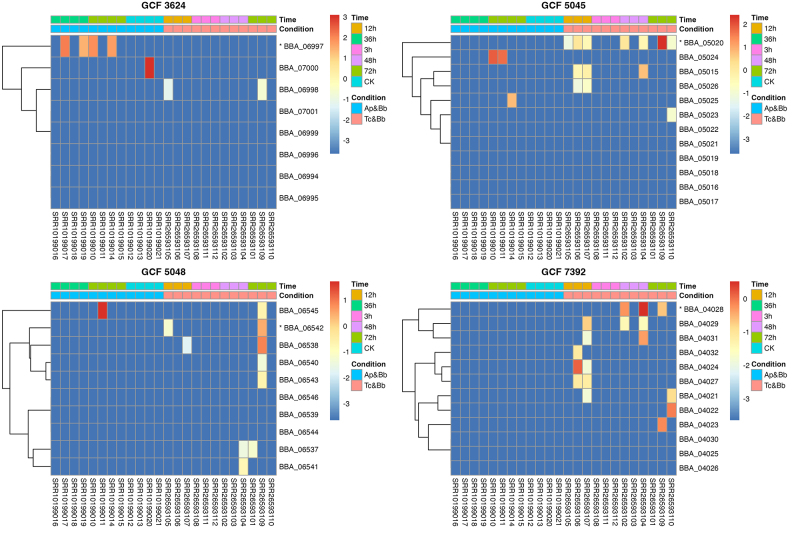
Expression patterns of *B. bassiana* biosynthetic gene clusters (BGCs) within gene cluster families (GCFs) during insect infection. The heatmaps display quantitative gene expression levels derived from Variance Stabilizing Transformation (VST) of read counts for genes within *B. bassiana* (Bb) BGCs. Data represent expression across various time points of infection in two insect hosts: the pea aphid (*Acyrthosiphon pisum* - Ac) and the red flour beetle (*Tribolium castaneum* - Tc). RNA-Seq data were retrieved from the Sequence Read Archive (SRA) and aligned to the *B. bassiana* ARSEF 2860 genome. Each panel represents one of the evaluated BGCs from each prioritized GCF. The columns indicate samples grouped by time point and host, and rows represent individual genes within the BGC. The color scale indicates relative expression intensity, where red signifies robust induction during infection and blue represents lower expression levels typically associated with control or saprophytic conditions. Asterisks (*) denote backbone biosynthetic genes.

Fungi from the genus *Beauveria* are widely used for the biological control of agricultural pests. To assess whether the identified GCFs harbor BGCs potentially associated with virulence, we analyzed the expression of each gene within these BGCs using RNA-Seq data from a study that investigated the infection of the pea aphid (*Acyrthosiphon pisum*) and the red flour beetle (*Tribolium castaneum*) by *B. bassiana* at different time points. After retrieving the data from the SRA and aligning it to the *B. bassiana* ARSEF 2860 genome, gene counts were normalized using the DESeq2 package, and the expression levels of each *B. bassiana* BGC within these GCFs were plotted. Gene expression was quantified using the Variance Stabilizing Transformation (VST) to ensure statistical stability across the various infection time points and hosts. This robust normalization revealed that four of the evaluated BGCs exhibited significant upregulation under infection conditions relative to saprophytic controls, with backbone genes such as BBA_05020 (GCF 5045) and BBA_04028 (GCF 7392) showing the highest induction magnitudes (Figure 7), which suggesting a potential role in virulence in at least one of the hosts analyzed.

The highlighted GCFs, which encode enzymes implicated in virulence such as NRPS (GCF 5045, backbone gene BBA_05020) and Hybrids (GCF 7392, backbone gene BBA_04028), exhibit potential upregulation under infection conditions. This strongly suggests that their metabolic products play a significant role in the fungus-host interaction, making them prime candidates for further analysis to elucidate their precise functions in insect pathogenicity. 

## Discussion

Numerous studies have demonstrated the significance of filamentous fungi and the potential applications of their secondary metabolites. Consequently, the study of their biosynthetic gene clusters (BGCs) is fundamental, as they are crucial for diverse processes, including biological associations and infections. Our analysis of hundreds of fungal genomes revealed that most gene cluster families (GCFs) in the order Hypocreales are genus-specific or orphans (singletons), a pattern previously observed in bacterial and lichens BGCs (Meesil et al., 2023; Singh et al., 2024). This genomic distribution reflects a distinctive evolutionary pattern that enables fungi to inhabit and dominate a wide range of specific ecological niches, facilitating unique adaptations to diverse environments (Naranjo-Ortiz and Gabaldón, 2019). Coevolutionary studies demonstrate that fungi develop specific adaptations, enabling them to adjust to their environment while also specializing for distinct hosts. This evolutionary pressure can result in diverse interactions, from mutualism to specialized parasitism (Zaccaron and Stergiopoulos, 2025). For example, some entomopathogenic fungi form mutualistic associations by developing specific genes to interact with various plant species. In contrast, parasitic specialization involves the evolution of toxins specific to different insect species, the development of specialized transmission mechanisms, and the adaptation of strategies to control growth within the host, as exemplified by entomopathogenic fungi when they infect insects (Zhang et al., 2020). In line with these observations, a recent survey in near 85,000 known species from Pezizomycotina revealed the capacity to produce and estimated 4 million SMs derived from 870 thousand to 2.7 million gene cluster families (Riedling and Rokas, 2025; Zaccaron and Stergiopoulos, 2025). 

The observed lognormal distribution of GCFs points to restricted expansion of conserved BGCs among species, mainly because of strong selective pressure that would allow BGCs diversification and subsequently constraining GCF expansion. This finding is consistent with the absence of extreme superfamilies, and suggests that successful fungal evolution selects for specialized biosynthetic capabilities over broad, generalist strategies (Aghdam and Brown 2021). The prevalence of orphan and singleton GCFs indicates a dual adaptative strategy, balancing the stability of conserved genus-level biosynthetic pathways with the flexibility afforded by rapid innovation through the independent creation of GCFs. Recent research suggests this lack of broad conservation at larger evolutionary distances is driven by evidence of high turnover among BGCs, which contributes to the observed chemodiversity within the largest classes of fungi (Gluck-Thaler et al., 2020). This combination of stability and flexibility likely underpins the ecological success of fungi in diverse habitats (Muggia et al., 2020).

Although most GCFs were genus-specific, our attention was drawn to 16 GCFs that were present in at least three distinct entomopathogenic genera. The BGCs within these shared families are thus prime candidates for functional analysis, based on the hypothesis that they are crucial for virulence and fungal pathogenesis (Quesada-Moraga et al., 2023). Among the 16 GCFs of interest, GCF_5048 particularly stands out for being present in four distinct entomopathogenic genera, rendering its constituent BGCs high-priority targets for functional characterization. Functional priorization strategies should focus on genes reported as upregulated under relevant conditions in the literature. Since the backbone gene of a BGC determines the core structure of its metabolic product, its high expression during infection renders it a prime target for functional analysis (Wang et al., 2021).

Such genes are therefore strong candidates for studies aimed at the discovery of new natural products (Riedling et al., 2024). The *B. bassiana* backbone genes BBA_05020 (GCF 5045) and BBA_04028 (GCF 7392) were upregulated during infection of *T. castaneum* at 72h and 48h, respectively. Further characterization identified BBA_05020 as SIDC, a non-ribosomal peptide synthetase that orchestrates a complex set of at least 11 siderophores, including ferricrocin and fusarinine C. This biosynthetic pathway is critical for maintaining iron homeostasis during the infection process, specifically facilitating fungal penetration of the host cuticle. Siderophore assembly catalyzed by SIDC and related genes like SIDD is also essential for conidial germination under the iron-deficient conditions encountered at the host interface (Sun et al., 2024). The significant upregulation of GCF 5045 during infection (Figure 7) underscores the vital role of iron metabolism in entomopathogenic virulence. The upregulation during infection was not limited to the backbone genes, other genes within the GCFs highlighted in Figure 7 also showed increased expression. This positions them as promising targets for functional characterization of their roles in the fungus-host interaction. Such host-responsive induction of secondary metabolite pathways aligns with previous findings in entomopathogenic fungi (Zhang et al., 2020). Moreover, the differential expression of genes within these clusters, particularly the backbone genes, confirms their metabolic activity and strongly implicates them in host adaptation, marking them as prime candidates for genetic manipulation to improve biocontrol strategies. Notably, the BGCs from *B. bassiana* ARSEF 2860 highlighted in Figure 7 are currently not deposited in the MIBiG repository. 

Furthermore, while *B. bassiana* possesses several BGCs that are known to produce various compounds, the overall number of characterized clusters is still limited. This highlights the need for targeted studies to characterize the vast number of unannotated and undeposited BGCs in this species in the MIBiG database. Regarding the specific GCFs identified here, the upregulation of core genes within GCF 5045 and GCF 7392 provides evidence of the evolution of BGCs specialized for interactions with particular hosts. This finding, when considered alongside our results for the other entomopathogen-conserved GCFs, underscores the role of fungal specificity in dictating host choice and activating virulence factors. It implies that the fungal response is not uniform but is instead tailored to specific host-derived stimuli (Rinker et al., 2024). This result demonstrates a critical point where the presence of a fungal BGC doesn’t guarantee its expression or involvement in every pathogen context. This highlights the sophisticated regulatory mechanisms governing these clusters and emphasizes the need for deeper research into their specific activation triggers. 

Bioinformatics tools and their applications have advanced the study of fungal BGCs, a field that deserves greater attention. While our *in silico* analyses yielded significant hypotheses, experimental validation is essential. Future research should involve wet-lab techniques (for example, CRISPR-Cas9, metabolomics) and broader taxonomic surveys to assess the entomopathogenic specificity of these clusters. It must be noted that these computational predictions are influenced by numerous parameters and demand meticulously curated, high-quality genomic data for accuracy, underscoring the necessity of experimental verification. Building on this, our study reinforces that orphan and singleton BGCs represent a vast, underexplored reservoir of biosynthetic potential. Focus on investigating them could pave the way for discovering new bioactive compounds of significant biotechnological value. 

## Supplementary material

The following online material is available for this article:

Table S1 - Genome sequences analyzed, size, and BGC counts.

Table S2 - Power law analysis results.

Table S3 - Exclusive GCFs from entomopathogenic genera.

Figure S1 - Identity analysis of GCF 2292.

Figure S2 - Identity analysis of GCF 3615.

Figure S3 - Identity analysis of GCF 3624.

Figure S4 - Identity analysis of GCF 3634.

Figure S5 - Identity analysis of GCF 3644.

Figure S6 - Identity analysis of GCF 4693.

Figure S7 - Identity analysis of GCF 5045.

Figure S8 - Identity analysis of GCF 5048.

Figure S9 - Identity analysis of GCF 5052.

Figure S10 - Identity analysis of GCF 5402.

Figure S11 - Identity analysis of GCF 7082.

Figure S12 - Identity analysis of GCF 7102.

Figure S13 - Identity analysis of GCF 7120.

Figure S14 - Identity analysis of GCF 7366.

Figure S15 - Identity analysis of GCF 7384.

Figure S16 - Identity analysis of GCF 7392.
