# Supplementary material for: Conserved gene clusters in entomopathogenic filamentous fungi
Source: Genet Mol Biol. 2026 Apr 17;49(1):e20250168. doi: 10.1590/1678-4685-GMB-2025-0168 (PMC13123250; doi:10.1590/1678-4685-GMB-2025-0168)
Supplement: Table S1 - [file 1415-4757-GMB-49-1-e20250168-s1.pdf]

Genetics and Molecular Biology
https://doi.org/10.1590/1678-4685-GMB-2025-0168

Supplementary Material to “Conserved gene clusters in entomopathogenic filamentous fungi”

Table S1 - Genome sequences analyzed. size. and BGC counts.

GCA_ID	Organism	Genome Size(bp)	BUSCO (%)	BGC
**GCA_013085055.1**	*Fusarium oxysporum Fo47*	50358849	0.996	47
**GCA_020647865.1**	*Trichoderma asperellum*	37545380	0.994	47
**GCA_020647795.1**	*Trichoderma atroviride*	37300646	0.994	39
**GCA_000687475.2**	*Ustilaginoidea virens*	37060086	0.989	23
**GCA_003814445.1**	*Epichloe festucae Fl1*	34970730	0.988	35
**GCA_016952355.1**	*Fusarium culmorum*	37519305	0.997	39
**GCA_013426205.1**	*Metarhizium brunneum*	37771916	0.995	66
**GCA_022605165.1**	*Purpureocillium takamizusanense*	35574015	0.997	32
**GCA_019565615.1**	*Trichoderma simmonsii*	40078385	0.993	61
**GCA_900079805.1**	*Fusarium fujikuroi IMI 58289*	43832314	0.996	49
**GCA_000149555.1**	*Fusarium verticillioides 7600*	41791161	0.996	49
**GCA_001653235.2**	*Pochonia chlamydosporia 170*	44215803	0.996	54
**GCA_019609905.1**	*Fusarium poae*	43859651	0.996	43
**GCA_000151355.1**	*Fusarium vanettenii 77-13-4*	51286497	0.997	37
**GCA_000167675.2**	*Trichoderma reesei QM6a*	33395713	0.99	32
**GCA_003025095.1**	*Trichoderma harzianum CBS 226.95*	40980648	0.993	56
**GCA_001050175.1**	*Trichoderma parareesei*	32070663	0.983	32
**GCA_019669845.1**	*Emericellopsis atlantica*	27300990	0.986	35
**GCA_000814975.1**	*Metarhizium hybridum*	38504274	0.995	64
**GCA_003946995.1**	*Fusarium oligoseptatum*	48655532	0.996	40
**GCA_013396195.1**	*Fusarium tjaetaba*	43066134	0.991	51
**GCA_012932015.1**	*Fusarium acutatum*	43327976	0.995	50
**GCA_013396175.1**	*Fusarium denticulatum*	43161494	0.991	56
**GCA_013396205.1**	*Fusarium mundagurra*	49121928	0.991	53
**GCA_013396025.1**	*Fusarium phyllophilum*	43389320	0.986	55
**GCA_012932025.1**	*Fusarium austroafricanum*	45682287	0.986	58
**GCA_013781345.1**	*Fusarium coicis*	42721782	0.982	51
**GCA_013396165.1**	*Fusarium globosum*	44594533	0.979	59
**GCA_013395995.1**	*Fusarium pseudoanthophilum*	42829214	0.987	53
**GCA_018360045.1**	*Claviceps capensis*	27728898	0.979	28
**GCA_018360065.1**	*Claviceps pazoutovae*	27795191	0.977	27
**GCA_013266185.1**	*Fusarium sarcochroum*	46449918	0.983	47
**GCA_018360055.1**	*Claviceps monticola*	27635999	0.98	27
**GCA_000280675.1**	*Beauveria bassiana ARSEF 2860*	33693936	0.991	44
**GCA_000225605.1**	*Cordyceps militaris CM01*	32268578	0.986	30
**GCA_012934285.1**	*Ophiocordyceps sinensis*	110880992	0.987	42
**GCA_900067095.1**	*Fusarium proliferatum ET1*	45210324	0.997	57
**GCA_013305495.1**	*Metarhizium anisopliae*	42848098	0.996	59
**GCA_019434415.1**	*Metarhizium acridum*	44714781	0.986	43
**GCA_000187425.2**	*Metarhizium robertsii ARSEF 23*	41656800	0.994	68
**GCA_000170995.2**	*Trichoderma virens Gv29-8*	39022666	0.992	59
**GCA_000260195.2**	*Fusarium odoratissimum NRRL 54006*	46553780	0.997	44
**GCA_020744515.1**	*Fusarium venenatum*	43693088	0.995	55
**GCA_003025115.1**	*Trichoderma citrinoviride*	33215161	0.964	34

GCA_ID	Organism	Genome Size(bp)	BUSCO (%)	BGC
**GCA_020744135.1**	*Cordyceps fumosorosea ARSEF 2679*	37452605	0.996	39
**GCA_001636725.1**	*Ilyonectria destructans*	33485962	0.988	34
**GCA_020740775.1**	*Trichoderma asperelloides*	71715727	0.996	61
**GCA_020360975.1**	*Hirsutella rhossiliensis*	81749301	0.994	84
**GCA_020744425.1**	*Dactylonectria macrodidyma*	77328613	0.995	58
**GCA_020744475.1**	*Fusarium redolens*	52563400	0.995	49
**GCA_021066465.1**	*Akanthomyces lecanii RCEF 1005*	36270279	0.993	47
**GCA_001636795.1**	*Tolypocladium ophioglossoides CBS 100239*	35594087	0.992	34
**GCA_001189435.1**	*Ilyonectria robusta*	31245833	0.99	34
**GCA_021365365.1**	*Trichoderma gamsii*	59648482	0.995	52
**GCA_001481775.2**	*Hirsutella minnesotensis 3608*	37908251	0.994	43
**GCA_000956045.1**	*Fusarium flagelliforme*	51111088	0.977	89
**GCA_020744385.1**	*Trichoderma gracile*	40328194	0.996	43
**GCA_020002365.1**	*Ophiocordyceps camponoti-floridani*	34062918	0.992	28
**GCA_012980515.1**	*Stachybotrys elegans*	30193175	0.964	31
**GCA_020746835.1**	*Dactylonectria estremocensis*	43467584	0.988	75
**GCA_020744375.1**	*Thelonectria olida*	64663708	0.996	43
**GCA_020736575.1**	*Fusarium mangiferae*	52862545	0.993	54
**GCA_900044065.1**	*Sarocladium implicatum*	46292722	0.996	51
**GCA_021176775.1**	*Metarhizium album ARSEF 1941*	30180565	0.984	34
**GCA_000804445.1**	*Trichoderma guizhouense*	30449065	0.991	31
**GCA_002022785.1**	*Cylindrodendrum hubeiense*	38329450	0.994	56
**GCA_014621425.1**	*Geosmithia morbida*	48811092	0.977	55
**GCA_012550715.1**	*Metarhizium guizhouense ARSEF 977*	26543684	0.967	15
**GCA_000814955.1**	*Moelleriella libera RCEF 2490*	43465197	0.994	66
**GCA_001636675.1**	*Stachybotrys chlorohalonatus IBT 40285*	30871875	0.983	30
**GCA_000732775.1**	*[Torrubiella] hemipterigena*	34390047	0.986	51
**GCA_000825705.1**	*Clonostachys solani*	28476206	0.979	37
**GCA_902141235.2**	*Metarhizium humberi*	54492124	0.989	67
**GCA_020102295.1**	*Trichoderma lentiforme*	38586956	0.994	70
**GCA_011066345.1**	*Clonostachys byssicola*	38315448	0.993	62
**GCA_902006505.2**	*Fusarium chuoi*	55062982	0.991	72
**GCA_022627125.1**	*Fusarium subglutinans*	45424172	0.996	38
**GCA_013396075.1**	*Fusarium heterosporum*	44177197	0.997	52
**GCA_013396295.1**	*Fusarium duplospermum*	35674141	0.984	44
**GCA_003946985.1**	*Fusarium coffeatum*	47510963	0.994	42
**GCA_003316985.1**	*Fusarium mexicanum*	37769732	0.994	37
**GCA_013396015.1**	*Fusarium gaditjirri*	43957871	0.992	60
**GCA_013266175.1**	*Fusarium anthophilum*	41888603	0.993	54
**GCA_013364935.1**	*Fusarium ambrosium*	45752990	0.989	61
**GCA_003947045.1**	*Fusarium pseudocircinatum*	49019568	0.991	39
**GCA_013396035.1**	*Claviceps arundinis*	43320561	0.989	51
**GCA_018360175.1**	*Fusarium kuroshium*	30330584	0.981	32
**GCA_003698175.1**	*Fusarium graminum*	46557493	0.946	43
**GCA_013266165.1**	*Ophiocordyceps camponoti-leonardi (nom. inval.)*	34983573	0.989	44
**GCA_003339455.1**	*Claviceps maximensis*	37909978	0.966	34
**GCA_018360255.1**	*Fusarium napiforme*	32978932	0.986	21
**GCA_013396005.1**	*Fusarium beomiforme*	42092515	0.983	50
**GCA_002980475.2**	*Ophiocordyceps camponoti-saundersi (nom. inval.)*	46521357	0.992	56
**GCA_003339415.1**	*Ophiocordyceps australis*	49265285	0.969	34
**GCA_002591415.1**	*Fusarium floridanum*	23324075	0.966	39
**GCA_003947005.1**	*Stylonectria norvegica*	47357966	0.961	40
**GCA_014621405.1**	*Claviceps humidiphila*	35092433	0.946	33
**GCA_018360465.1**	*Fusarium bulbicola*	31161740	0.978	29
**GCA_013758895.1**	*Fusarium zealandicum*	43605813	0.992	55

GCA_ID	Organism	Genome Size(bp)	BUSCO (%)	BGC
**GCA_013266195.1**	*Claviceps spartinae*	33536579	0.975	33
**GCA_018360215.1**	*Claviceps africana*	29308890	0.975	33
**GCA_018360145.1**	*Tolypocladium paradoxum*	37712632	0.947	24
**GCA_002916505.1**	*Fusarium decemcellulare*	27610157	0.978	32
**GCA_013266205.1**	*Claviceps digitariae*	53686487	0.953	71
**GCA_018360205.1**	*Ophiocordyceps polyrhachis-furcata BCC 54312*	33417373	0.969	25
**GCA_001633055.2**	*Claviceps cyperi*	43252821	0.969	29
**GCA_018360075.1**	*Fusarium albosuccineum*	26646729	0.974	25
**GCA_012931995.1**	*Ophiocordyceps camponoti-rufipedis*	50867201	0.976	62
**GCA_002591395.1**	*Tolypocladium capitatum*	21899844	0.92	36
**GCA_002901185.1**	*Ophiocordyceps unilateralis*	22985095	0.977	32
**GCA_001272575.2**	*Claviceps citrina*	23913698	0.968	35
**GCA_018360365.1**	*Claviceps sorghi*	43472126	0.92	22
**GCA_018360135.1**	*Claviceps lovelessii*	35635695	0.924	26
**GCA_018360185.1**	*Cordyceps militaris*	41063740	0.92	29
**GCA_008080495.1**	*Fusarium pseudograminearum*	33618380	0.989	34
**GCA_016952305.1**	*Trichoderma virens*	37805715	0.996	39
**GCA_020647635.1**	*Trichoderma virens FT-333*	40979523	0.993	55
**GCA_020647705.1**	*Fusarium fujikuroi*	41418917	0.992	53
**GCA_009663055.1**	*Fusarium fujikuroi*	43722320	0.995	47
**GCA_009663095.1**	*Fusarium fujikuroi*	43737213	0.996	46
**GCA_009663115.1**	*Fusarium oxysporum f. sp. lycopersici 4287*	45614948	0.979	44
**GCA_000149955.2**	*Fusarium oxysporum f. sp. conglutinans*	61386934	0.98	50
**GCA_014154955.1**	*Fusarium oxysporum f. sp. cepae*	67983296	0.996	43
**GCA_003615085.1**	*Fusarium verticillioides*	53425788	0.996	51
**GCA_003316975.2**	*Fusarium poae*	42535067	0.996	43
**GCA_001675295.1**	*Metarhizium robertsii*	46317270	0.996	47
**GCA_000591435.1**	*Fusarium circinatum*	40317276	0.994	68
**GCA_013396185.1**	*Trichoderma longibrachiatum ATCC 18648*	42547896	0.991	47
**GCA_003025155.1**	*Fusarium longipes*	32238325	0.897	27
**GCA_003012285.1**	*Fusarium oxysporum*	35314544	0.986	39
**GCA_020744355.1**	*Fusarium oxysporum*	55719217	0.997	48
**GCA_020744455.1**	*Fusarium oxysporum*	52242347	0.993	47
**GCA_900096695.1**	*Fusarium oxysporum*	49085590	0.997	46
**GCA_003615185.1**	*Fusarium oxysporum*	50292220	0.995	48
**GCA_003615165.1**	*Fusarium oxysporum*	50515499	0.996	48
**GCA_003615115.1**	*Fusarium oxysporum*	54787818	0.996	53
**GCA_013363075.1**	*Fusarium oxysporum f. sp. cubense*	46979197	0.929	54
**GCA_005930515.1**	*Fusarium oxysporum f. sp. cubense*	51139495	0.996	46
**GCA_007994515.1**	*Fusarium oxysporum f. sp. conglutinans*	48588396	0.997	44
**GCA_018894095.1**	*Fusarium oxysporum Fo47*	72150645	0.996	47
**GCA_000271705.2**	*Fusarium oxysporum f. sp. cubense race 1*	49664628	0.996	46
**GCA_000350345.1**	*Fusarium oxysporum f. sp. matthiolae*	47657417	0.994	42
**GCA_020796175.1**	*Fusarium oxysporum f. sp. narcissi*	60310576	0.996	46
**GCA_004141715.1**	*Fusarium oxysporum f. sp. rapae*	57517562	0.995	51
**GCA_019157295.1**	*Fusarium oxysporum f. sp. raphani*	59760091	0.996	57
**GCA_019157275.1**	*Fusarium oxysporum f. sp. radicis-lycopersici 26381*	66822448	0.997	47
**GCA_000260155.3**	*Fusarium oxysporum f. sp. lycopersici MN25*	49359289	0.996	46
**GCA_000259975.2**	*Fusarium oxysporum f. sp. melonis 26406*	48637398	0.996	48
**GCA_000260495.2**	*Fusarium oxysporum f. sp. pisi HDV247*	54034280	0.997	53
**GCA_000260075.2**	*Fusarium oxysporum f. sp. vasinfectum 25433*	55188216	0.996	47
**GCA_000260175.2**	*Fusarium oxysporum f. sp. cepae*	52914414	0.997	47
**GCA_003615075.1**	*Fusarium oxysporum f. sp. cepae*	51009701	0.995	51
**GCA_003615095.1**	*Fusarium oxysporum f. sp. raphani 54005*	51407732	0.996	51
**GCA_000260235.2**	*Fusarium oxysporum f. sp. conglutinans race 2 54008*	53499362	0.996	45

GCA_ID	Organism	Genome Size(bp)	BUSCO (%)	BGC
**GCA_000260215.2**	*Fusarium graminearum*	53575352	0.996	53
**GCA_900492705.2**	*Fusarium graminearum*	36807931	0.988	44
**GCA_002352725.1**	*Fusarium graminearum*	36883775	0.997	46
**GCA_905359455.1**	*Fusarium graminearum*	36456942	0.996	45
**GCA_905332025.1**	*Fusarium graminearum*	36563540	0.996	44
**GCA_905332055.1**	*Fusarium graminearum*	37010759	0.977	44
**GCA_905359485.1**	*Fusarium graminearum*	37136041	0.996	47
**GCA_905359475.1**	*Fusarium graminearum*	36760321	0.996	44
**GCA_901446245.1**	*Fusarium graminearum*	36753730	0.996	45
**GCA_905359465.1**	*Beauveria bassiana*	36282076	0.986	45
**GCA_001682635.1**	*Beauveria bassiana*	35021612	0.991	41
**GCA_021365345.1**	*Beauveria bassiana*	35662634	0.991	41
**GCA_010099065.1**	*Beauveria bassiana*	35449500	0.989	38
**GCA_002871155.1**	*Beauveria bassiana D1-5*	36538394	0.992	36
**GCA_000770705.1**	*Trichoderma reesei RUT C-30*	36692259	0.96	44
**GCA_000513815.1**	*Trichoderma harzianum*	32681481	0.991	31
**GCA_002894145.1**	*Trichoderma harzianum*	38779109	0.993	57
**GCA_000988865.1**	*Trichoderma harzianum*	39721542	0.989	56
**GCA_002838845.1**	*Trichoderma asperellum*	40865014	0.988	57
**GCA_013423425.1**	*Fusarium fujikuroi*	36071795	0.995	39
**GCA_900096505.1**	*Fusarium fujikuroi*	43998436	0.996	53
**GCA_900096635.1**	*Fusarium fujikuroi*	44975616	0.996	49
**GCA_900096645.1**	*Fusarium fujikuroi*	45765444	0.996	48
**GCA_900096615.1**	*Fusarium fujikuroi*	44041582	0.996	49
**GCA_901677965.1**	*Fusarium fujikuroi*	46485183	0.996	48
**GCA_900096705.1**	*Fusarium fujikuroi*	46133096	0.996	50
**GCA_901677955.1**	*Fusarium fujikuroi*	45814433	0.995	52
**GCA_902702945.1**	*Fusarium fujikuroi*	46029076	0.989	37
**GCA_900096625.1**	*Fusarium fujikuroi*	45342496	0.996	43
**GCA_900096685.1**	*Fusarium fujikuroi*	46063609	0.996	49
**GCA_900096605.1**	*Fusarium fujikuroi*	44324837	0.989	53
**GCA_001023035.1**	*Fusarium fujikuroi*	43110459	0.988	50
**GCA_001023045.1**	*Fusarium fujikuroi*	43096072	0.966	48
**GCA_001023065.1**	*Ophiocordyceps sinensis CO18*	43499819	0.959	57
**GCA_000448365.1**	*Fusarium proliferatum*	78515811	0.762	26
**GCA_017309895.1**	*Fusarium proliferatum*	43842875	0.996	54
**GCA_017309875.1**	*Fusarium proliferatum*	45293945	0.996	54
**GCA_022627135.1**	*Fusarium proliferatum*	44982388	0.996	38
**GCA_900029915.1**	*Fusarium proliferatum*	43166078	0.996	54
**GCA_003290285.1**	*Fusarium proliferatum*	45499559	0.996	56
**GCA_003615215.1**	*Fusarium proliferatum*	45689467	0.997	58
**GCA_017309865.1**	*Metarhizium anisopliae*	48779490	0.996	50
**GCA_000739145.1**	*Metarhizium anisopliae BRIP 53284*	38477109	0.991	62
**GCA_000426985.1**	*Metarhizium anisopliae BRIP 53293*	38088637	0.981	67
**GCA_000426965.1**	*Metarhizium acridum*	38672492	0.99	64
**GCA_019434395.1**	*Ustilaginoidea virens*	39252651	0.573	49
**GCA_000965225.2**	*Purpureocillium lilacinum*	33567624	0.975	17
**GCA_003144605.1**	*Purpureocillium lilacinum*	37598801	0.996	36
**GCA_001653205.1**	*Clonostachys rosea*	38140521	0.997	35
**GCA_902085965.1**	*Fusarium odoratissimum*	53069127	0.97	75
**GCA_910393935.1**	*Fusarium equiseti*	37882472	0.995	34
**GCA_000350365.1**	*Fusarium avenaceum*	52926277	0.997	33
**GCA_020744115.1**	*Fusarium avenaceum*	42232206	0.995	59
**GCA_000769215.1**	*Fusarium avenaceum*	41541670	0.996	62
**GCA_018282135.1**	*Fusarium tricinctum*	41192406	0.996	49

GCA_ID	Organism	Genome Size(bp)	BUSCO (%)	BGC
**GCA_019425555.1**	*Fusarium venenatum*	37570916	0.996	39
**GCA_006981985.1**	*Metarhizium rileyi*	34933242	0.991	42
**GCA_006981975.1**	*Metarhizium rileyi*	34972450	0.99	42
**GCA_001636745.1**	*Cordyceps javanica*	32013981	0.994	32
**GCA_007866325.1**	*Cordyceps javanica*	31808756	0.993	30
**GCA_000769265.1**	*Hapsidospora chrysogenum ATCC 11550*	28563214	0.985	42
**GCA_003012315.1**	*Fusarium sporotrichioides*	37429079	0.992	47
**GCA_003033665.1**	*Fusarium culmorum*	38110313	0.997	40
**GCA_001306435.1**	*Neonectria ditissima*	45719213	0.992	43
**GCA_018296325.1**	*Fusarium xylarioides*	57004444	0.995	50
**GCA_018296245.1**	*Fusarium xylarioides*	60032360	0.995	51
**GCA_018296285.1**	*Fusarium xylarioides*	59292093	0.995	51
**GCA_018296265.1**	*Fusarium xylarioides*	61006077	0.995	51
**GCA_018296345.1**	*Fusarium xylarioides*	62280680	0.996	46
**GCA_018296305.1**	*Fusarium xylarioides*	63055833	0.995	44
**GCA_001653215.1**	*Akanthomyces lecanii*	32603708	0.818	28
**GCA_018360975.1**	*Claviceps purpurea*	30645442	0.98	30
**GCA_018360845.1**	*Claviceps purpurea*	30538565	0.98	32
**GCA_018360835.1**	*Claviceps purpurea*	30556244	0.978	33
**GCA_018360955.1**	*Claviceps purpurea*	30607588	0.98	33
**GCA_018360655.1**	*Claviceps purpurea*	30626138	0.979	29
**GCA_018360685.1**	*Claviceps purpurea*	30602050	0.975	29
**GCA_018360925.1**	*Claviceps purpurea*	30759852	0.98	31
**GCA_018360745.1**	*Claviceps purpurea*	30683236	0.98	28
**GCA_018360905.1**	*Claviceps purpurea*	30796132	0.981	30
**GCA_018360675.1**	*Claviceps purpurea*	30512801	0.98	29
**GCA_018360475.1**	*Claviceps purpurea*	30528026	0.979	28
**GCA_018360735.1**	*Claviceps purpurea*	30590494	0.981	33
**GCA_018360855.1**	*Claviceps purpurea*	30642461	0.979	29
**GCA_018360725.1**	*Claviceps purpurea*	30760348	0.978	28
**GCA_000347355.1**	*Claviceps purpurea 20.1*	32091443	0.982	27
**GCA_018360765.1**	*Claviceps purpurea*	30508064	0.979	30
**GCA_018360755.1**	*Claviceps purpurea*	30537121	0.98	30
**GCA_018360825.1**	*Claviceps purpurea*	30523917	0.979	31
**GCA_018360865.1**	*Claviceps purpurea*	30689426	0.982	31
**GCA_018360535.1**	*Claviceps purpurea*	30502172	0.977	29
**GCA_018360585.1**	*Claviceps purpurea*	30693778	0.978	27
**GCA_018360565.1**	*Claviceps purpurea*	30512745	0.973	30
**GCA_001481775.1**	*Trichoderma gamsii*	37970415	0.991	42
**GCA_003957675.1**	*Fusarium euwallaceae*	50472048	0.987	38
**GCA_001292635.1**	*Fusarium langsethiae*	37543021	0.989	46
**GCA_001278495.1**	*Escovopsis weberi*	27200907	0.97	22
**GCA_001636735.1**	*Beauveria brongniartii RCEF 3172*	32515992	0.989	40
**GCA_000732565.1**	*Stachybotrys chartarum IBT 40293*	36480513	0.989	58
**GCA_000730325.1**	*Stachybotrys chartarum IBT 7711*	36879702	0.989	59
**GCA_000732765.1**	*Stachybotrys chartarum IBT 40288*	36012651	0.961	57
**GCA_002894225.1**	*Fusarium nygamai*	50242135	0.996	53
**GCA_003012295.1**	*Fusarium flagelliforme*	39607924	0.987	41
**GCA_003012105.1**	*Trichoderma arundinaceum*	36872412	0.973	49
**GCA_020744335.1**	*Fusarium sp. MPI-SDFR-AT-0072*	48370669	0.997	52
**GCA_020744155.1**	*Ilyonectria sp. MPI-CAGE-AT-0026*	62828295	0.995	59
**GCA_022627095.1**	*Fusarium sp. FIESC RH6*	39998308	0.996	35
**GCA_022627115.1**	*Fusarium annulatum*	44219330	0.996	41
**GCA_013396095.1**	*Fusarium sp. NRRL 52700*	42984365	0.996	49
**GCA_902077795.2**	*Clonostachys rhizophaga*	59593020	0.989	74

GCA_ID	Organism	Genome Size(bp)	BUSCO (%)	BGC
**GCA_022627105.1**	*Fusarium chuoi*	44398943	0.996	37
**GCA_013364965.1**	*Fusarium austroamericanum*	36846346	0.99	44
**GCA_013396255.1**	*Fusarium sp. NRRL 25303*	43724536	0.993	62
**GCA_018360085.1**	*Claviceps arundinis*	30592454	0.977	31
**GCA_003947015.1**	*Fusarium sp. AF-6*	43054081	0.971	34
**GCA_018360325.1**	*Claviceps aff. humidiphila group G2b*	31203652	0.982	27
**GCA_018360525.1**	*Claviceps sp. Clav50 group G5*	28784330	0.979	26
**GCA_018360435.1**	*Claviceps sp. LM218 group G6*	31138742	0.978	28
**GCA_018360355.1**	*Claviceps sp. Clav32 group G5*	28658995	0.979	27
**GCA_018360595.1**	*Claviceps sp. LM219 group G6*	30771931	0.974	28
**GCA_018360445.1**	*Claviceps sp. LM220 group G6*	30889598	0.976	30
**GCA_018361025.1**	*Claviceps aff. purpurea*	29595454	0.981	30
**GCA_018360275.1**	*Claviceps sp. LM458 group G5*	28432374	0.975	25
**GCA_001654555.2**	*Fusarium agapanthi*	41300597	0.981	41
**GCA_018360575.1**	*Claviceps sp. LM454 group G7*	31185114	0.975	31
**GCA_018360345.1**	*Claviceps sp. LM84 group G4*	28940137	0.973	31
**GCA_013266265.1**	*Fusarium sp. NRRL 66182*	48024423	0.974	56
**GCA_018360385.1**	*Claviceps sp. LM78 group G4*	28765009	0.966	32
**GCA_018360375.1**	*Claviceps sp. LM77 group G4*	28694029	0.969	31
**GCA_014764975.1**	*Fusarium sp. DS 682*	56063992	0.974	59
**GCA_002591385.1**	*Cordyceps sp. RAO-2017*	32302071	0.846	46
**GCA_002591405.1**	*Ophiocordyceps australis*	22189873	0.932	34
**GCA_900007375.1**	*Fusarium venenatum*	38581574	0.996	39
**GCA_000271745.2**	*Fusarium oxysporum NRRL 32931*	47906303	0.996	47
**GCA_020744495.1**	*Fusarium solani*	52932111	0.997	41
**GCA_003025105.1**	*Trichoderma asperellum CBS 433.97*	37464610	0.995	48
**GCA_000171015.2**	*Trichoderma atroviride IMI 206040*	36143664	0.993	43
**GCA_000187405.1**	*Metarhizium acridum CQMa 102*	39422329	0.995	41
**GCA_001653265.1**	*Purpureocillium lilacinum*	38534601	0.997	36
**GCA_000814965.1**	*Metarhizium brunneum ARSEF 3297*	37066166	0.995	61
**GCA_000814945.1**	*Metarhizium majus ARSEF 297*	42062993	0.995	61
