# Supplementary material for: Conserved gene clusters in entomopathogenic filamentous fungi
Source: Genet Mol Biol. 2026 Apr 17;49(1):e20250168. doi: 10.1590/1678-4685-GMB-2025-0168 (PMC13123250; doi:10.1590/1678-4685-GMB-2025-0168)
Supplement: Table S2 - [file 1415-4757-GMB-49-1-e20250168-s2.pdf]

Genetics and Molecular Biology
https://doi.org/10.1590/1678-4685-GMB-2025-0168

Supplementary Material to “Conserved gene clusters in entomopathogenic filamentous fungi”

Table S2 - Power law analysis results.

Class	Obs	PL_alpha	PL_xmin	LN_meanlog	LN_sdlog	Test_Statistic	P_Value	Better_Model
**NRPS**	1293	5.93	34	1.14	0.74	0.20	0.83	No significant difference
**Others**	636	2.02	1	-6.57	2.93	-4.37	1.23e-5	Lognormal
**PKS-NRP_Hybrids**	378	1.79	1	-0.29	1.58	-3.75	1.76e-4	Lognormal
**PKSI**	556	4.31	24	2.73	0.53	-0.70	0.48	No significant difference
**PKSother**	478	1.79	1	-0.41	1.64	-4.01	5.92e-5	Lognormal
**RiPPs**	135	2.15	1	-0.82	1.33	-1.92	0.05	No significant difference
**Terpene**	433	2.09	5	1.68	1.06	-2.56	0.01	Lognormal
