# Supplementary material for: Conserved gene clusters in entomopathogenic filamentous fungi
Source: Genet Mol Biol. 2026 Apr 17;49(1):e20250168. doi: 10.1590/1678-4685-GMB-2025-0168 (PMC13123250; doi:10.1590/1678-4685-GMB-2025-0168)
Supplement: Table S3 - [file 1415-4757-GMB-49-1-e20250168-s3.pdf]

Genetics and Molecular Biology
https://doi.org/10.1590/1678-4685-GMB-2025-0168

Supplementary Material to “Conserved gene clusters in entomopathogenic filamentous fungi”

Table S3 - Exclusive GCFs from entomopathogenic genera.

GCF	Genus_1	Genus_2	Genus_3	Genus_4
**2292**	*Cordyceps*	*Beauveria*	*Akanthomyces*	*NA*
**3624**	*Beauveria*	*Cordyceps*	*Akanthomyces*	*NA*
**3634**	*Cordyceps*	*Beauveria*	*Akanthomyces*	*NA*
**3644**	*Cordyceps*	*Beauveria*	*Akanthomyces*	*NA*
**4693**	*Cordyceps*	*Beauveria*	*Akanthomyces*	*NA*
**5045**	*Cordyceps*	*Beauveria*	*Akanthomyces*	*NA*
**5052**	*Beauveria*	*Cordyceps*	*Akanthomyces*	*NA*
**7082**	*Beauveria*	*Akanthomyces*	*Cordyceps*	*NA*
**7102**	*Hirsutella*	*Akanthomyces*	*Cordyceps*	*NA*
**7384**	*Beauveria*	*Akanthomyces*	*Cordyceps*	*NA*
**7392**	*Cordyceps*	*Beauveria*	*Akanthomyces*	*NA*
**3615**	*Cordyceps*	*Beauveria*	*Akanthomyces*	*NA*
**7120**	*Hirsutella*	*Metarhizium*	*Purpureocillium*	*NA*
**7366**	*Beauveria*	*Akanthomyces*	*Cordyceps*	*NA*
**5048**	*Cordyceps*	*Beauveria*	*Hirsutella*	*Akanthomyces*
**5402**	*Cordyceps*	*Beauveria*	*Akanthomyces*	*NA*
