# Supplementary material for: Conserved gene clusters in entomopathogenic filamentous fungi
Source: Genet Mol Biol. 2026 Apr 17;49(1):e20250168. doi: 10.1590/1678-4685-GMB-2025-0168 (PMC13123250; doi:10.1590/1678-4685-GMB-2025-0168)
Supplement: Figure S11 - [file 1415-4757-GMB-49-1-e20250168-s14.pdf]

Genetics and Molecular Biology
https://doi.org/10.1590/1678-4685-GMB-2025-0168

Supplementary Material to "Conserved gene clusters in entomopathogenic filamentous fungi"

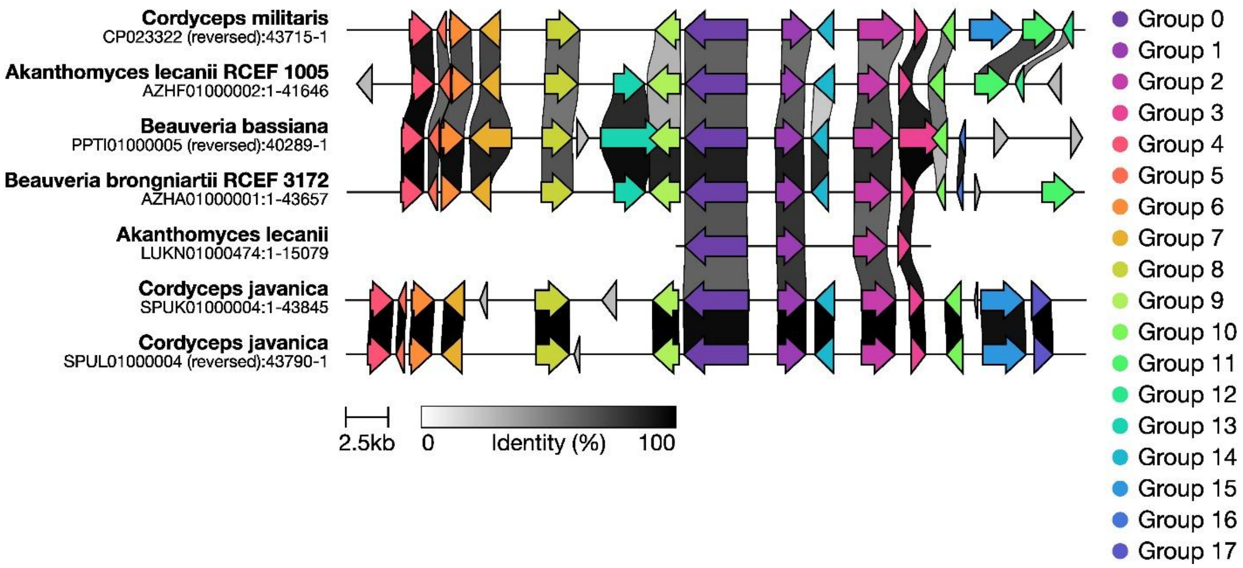

Figure S11 - Identity analysis of GCF 7082.
